# Monitoring of Alarm Reactions of Red Deer (*Cervus elaphus*) in a Captive Population in Paneveggio Pale di San Martino Natural Park

**DOI:** 10.3390/ani13050903

**Published:** 2023-03-01

**Authors:** Sara Moscatelli, Alessandro Malfatti, Federico Maria Tardella, Cesare Pacioni, Daniele Catorci, Paola Scocco

**Affiliations:** 1 International School of Advanced Studies, University of Camerino, Via Madonna delle Carceri 9, 62032 Camerino, Italy; 2 School of Biosciences and Veterinary Medicine, University of Camerino, Via Pontoni 5, 62032 Camerino, Italy; 3 Terrestrial Ecology Unit, Department of Biology, Faculty of Sciences, Ghent University, K. L. Ledeganckstraat 35, 9000 Ghent, Belgium; 4 Landscape Photographer, Mythlands

**Keywords:** red deer, alarm behavior, disturbance, vigilant attitudes, pasture restoring

## Abstract

**Simple Summary:**

After several years of inappropriate management, the pasture inside the enclosure for captive red deer in Paneveggio Pale di San Martino regional Park (TN, Italy) lost its nutritional value, due to the expansion of unpalatable tall grasses. Therefore, several measures to restore a suitable pasture composition were needed. The mowing activity represents a disturbance for the captive deer, which negatively affects the animals’ well-being. To establish the more appropriate times/days to perform activities inside the enclosure, we observed the alarm reactions and relative intensity of animals exposed to different visual stimuli presented inside and outside the enclosure. Some differences were highlighted between the males and the nursery (females and fawns) groups. Considering the deer biology and the studied location, the best months in which it would be possible to plan activities inside the enclosure are March, April (if the snow is not present) and August. Data elaboration suggests that the best day to perform activities inside the enclosure is Wednesday because the animals showed less sensitivity to disturbances; Tuesday and Thursday may also be considered additional suitable days.

**Abstract:**

The study analyzes red deer responses to disturbances during the day and different exposures to tourists, to establish the more appropriate times to carry out activities inside the Paneveggio deer enclosure. The alarm reactions of red deer were observed after presenting different types of visual stimuli inside and outside the fence, in order to answer some questions: Which stimuli produce the strongest reactions from the animals? Do animals differently react to stimuli presented outside and inside the fence? On which days and times are the animals more sensitive to disturbances? Are there different reactions between the males and females? The results suggest that the red deer adversely react to the disturbance at different degrees of intensity in relation to day, sex, tourist and where the stimuli are presented. It was observed that during the days with the highest tourist presence, the animals were particularly alarmed; discomfort accumulation produced the highest number of alarm reactions on Monday. For these reasons, it would be opportune to manage the pasture on Tuesday, Wednesday and Thursday, scheduled at specific times of day, preferably far from the estimated presence of tourists.

## 1. Introduction

The intensification of recreational activities in wild areas is resulting in an increase in disturbance to wildlife [1]. Human disturbance deriving from such activities is supposedly perceived by animals as a predation risk [2]. Therefore, animals tend to adapt to such disturbances by adopting a repertoire of behaviors aimed at avoiding predators and therefore reinforcing survival chances [3,4].

Due to deforestation and hunting activities, the alpine red deer (*Cervus elaphus*) population dramatically dropped at the beginning of the year 1800 and the species was considered extinct in the southern part of the Alps [5,6]. The species population has been experiencing a stable recovery since the 1950s as a result of spontaneous recolonization and reintroductions, with a peak of circa 3000 individuals estimated in 2008 [5].

For educational/didactic purposes, a captive population of red deer is kept at Paneveggio Pale di San Martino, which is part of the San Martino Natural Park (IT). Red deer are Intermediate Opportunistic Mixed feeders [7] with a forage selectivity related to food availability and quality [8,9]. The kept red deer receive a feed supply by the keepers; this causes the inappropriate exploitation of trophic resources inside the enclosure, which may lead to a loss of biodiversity [10] because the deer select the more palatable plants, causing an inhomogeneous disturbance to the grass species. In addition, the pasture inside the enclosure is not mowed periodically, favoring the spread of tall grasses such as *Bellardiochloa variegata, Brachypodium rupestre* and *Deschampsia cespitosa* [8,11,12]. 

Worsening in forage composition and increased abundance of high fibrous tall grasses could result in a decline in animals’ body condition, as a consequence of the increase in rumen keratinization degree, which limits the absorption of volatile fatty acids [13]. Indeed, the keratinized outer layers of the epithelium act as a protective layer for the mucosa from exogenous mechanical stimuli, while the volatile fatty acids obtained from cellulose digestion are metabolized by the deeper layers. Therefore, when the degree of keratinization increases, the absorptive ability decreases [9,14].

The study was requested by the Paneveggio Pale di San Martino National Park in order to plan the restoration activities needed to repristinate a suitable pasture for the animals. However, human presence inside the enclosure might produce stress in the animals and negatively affect their welfare. If an animal is not in a healthy state, prolonged stress may result in the triggering of adaptive processes, which can cause significant harm to its body state [15]. For this reason, any activity that may affect animal welfare should be timed based on the animals’ response to disturbances.

For this purpose, the alarm reactions of the red deer captive population inside the deer enclosure in Paneveggio Pale di San Martino Natural Park were observed after presenting different types of visual stimuli inside and outside the enclosure. The animals’ behaviors, such as vigilance, alert and alarm reactions, were observed since they may be linked with underlying “states”, such as fear and stress [16].

For this reason, we attempted to answer the following questions:

1. Which stimuli produce the strongest reactions from the animals?
2. Do animals differently react to stimuli presented outside and inside the enclosure?
3. What are the days and times in which the animals are more sensitive to disturbances?
4. Do different groups react to disturbances in different ways?

This information will be useful when planning the times and methods of each necessary intervention within the enclosure, from restoring the quality and composition of the pasture to maintenance interventions on the fence, but also for organizing entry for scientific or educational purposes.

Maintaining a low level of disturbance is also the most ethical choice when caring for captive animals in order to ensure “freedom from fear and distress”, one of the five fundamental freedoms formulated by Webster [17].

## 2. Materials and Methods

### 2.1. Study Area

**Figure 1:**
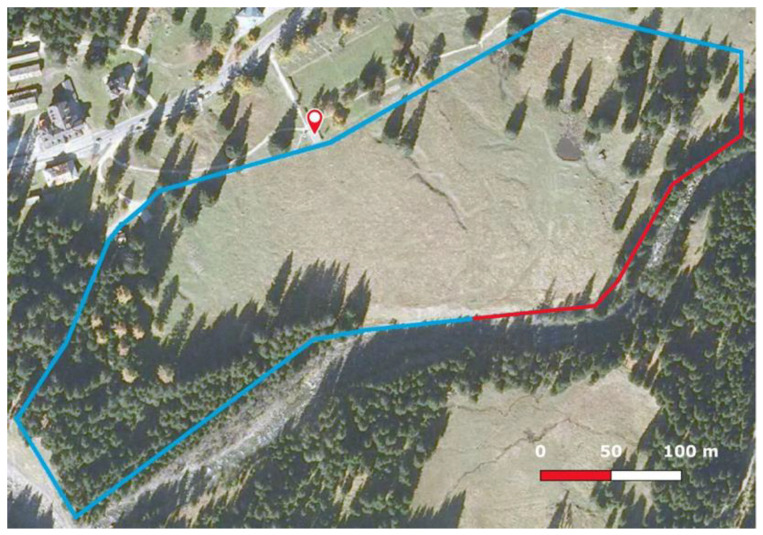
Aerial photo of the enclosure. On the left, a woodland is present, and the central part is an open pasture, and, on the right, a small lake is present. The blue line represents the easily walkable perimeter of the enclosure, while the red line corresponds to the less accessible path; mark shows the observation platform.

The study was conducted in Paneveggio Pale di San Martino Natural Park (IT), a protected area situated in the eastern part of the Alps. In particular, the captive red deer population inside the Paneveggio enclosure (46°18′24″ N, 11°44′24″ E) was observed. The enclosure is managed by the “Technical and Management Office of the State Forests Provincial Agency”, in Trento province, and has a 6-hectare extension (Figure 1); a quarter of it is composed of woodlands, while the remaining is pasture for the animals. A well-hidden lake is used by the animals to drink. The enclosure can accommodate up to a maximum of twenty animals.

**Figure 2:**
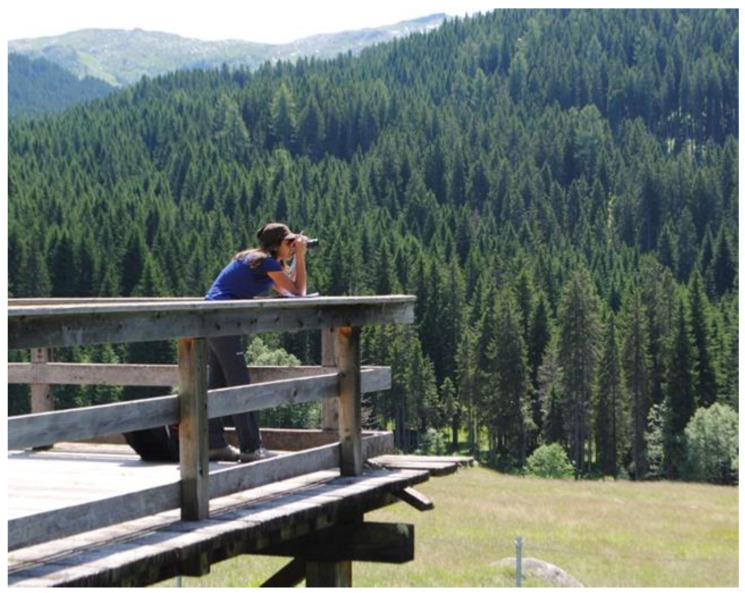
Observation platform.

Tourists are allowed to walk around the whole perimeter of the enclosure, with some easily walkable parts and some less accessible ones. Tourists are not allowed to enter the enclosure and their presence is variable depending on the day of the week, time of day and month. An observation platform is present, allowing the observation of the animals from a respectful distance (Figure 2).

### 2.2. Observation Procedure

During the study, the red deer population was composed of 18 individuals, 3 adult males, 10 adult females and 5 fawns. All deer present in the enclosure during the study were born in the enclosure and were fed only by their dams during the lactation period. The animals tended to separate into a nursery group (females and fawns) and a male group. The oldest of the males was the dominant one. The animals are subjected to intense exposure to tourists throughout the year, with higher incidences in the summer months. 

The study was performed during the touristic season of 2015, for 7 days during the month of July. Red deer alarm reactions were observed after presenting different stimuli in order to visually determine how the animals’ reactiveness changed throughout the day and the days of the week.

Three experienced observers, who had a good knowledge of the group of red deer, were present during the observation sessions, one presenting the stimulus and one hidden and recording the alarm reactions, while the third observer took photos and videos of the animal group in order to look for particular group patterns afterwards. The observers played the same roles for the whole study period.

Animals’ responses were observed with the naked eye by the observer presenting the stimulus, and with binoculars by the recording observer.

**Figure 3:**
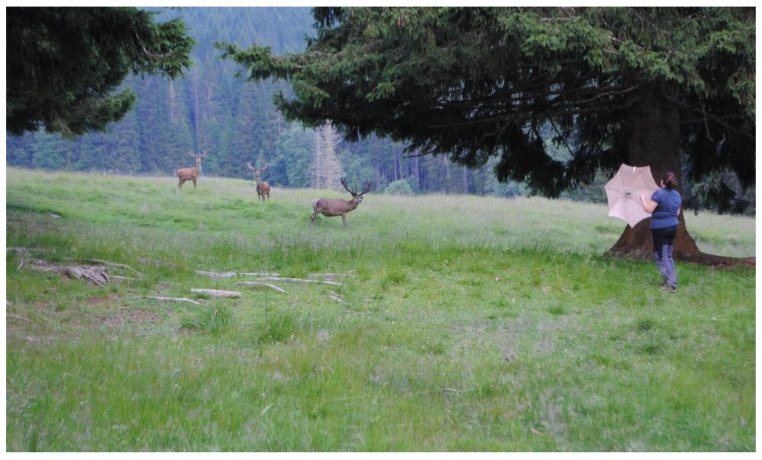
The stimulus of the umbrella opened inside the enclosure.

Following Hodgetts’s [18] study, stimuli that produced the greatest responses were selected, according to the possibility of reproducibility. Responses to three visual stimuli, i.e., (1) a person standing still (S) outside and inside the enclosure, (2) a person moving towards the animals (M) outside and inside the enclosure, (3) an umbrella opened (U) outside and inside (Figure 3) the enclosure, were examined (Table 1).

**Figure 4:**
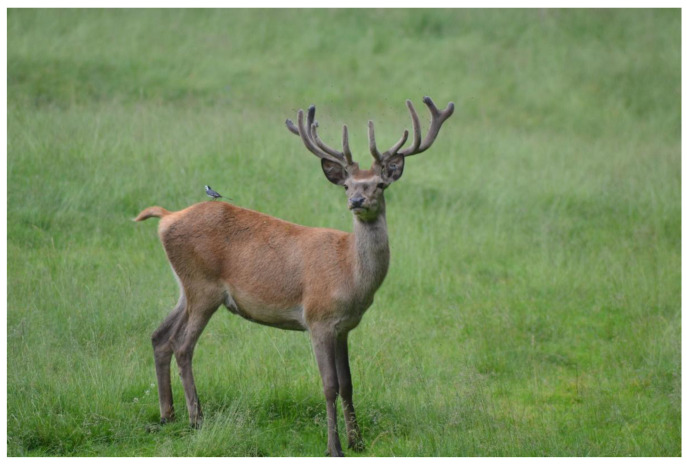
Alarmed subordinate male. The neck is prominent and the head is held high; the ears are directed toward the source of disturbance.

Alarm was detectable mostly by observing changing head and neck positions and ears’ movements; all these features were punctual and fast, but easily perceptible [4] (Figure 4). The observed alarm reactions were (i) head held high or parallel to the body, (ii) prominent neck, (iii) straight ears, (iv) ear twitching (rapid movement of both ears).

All the described alarm reactions were noticeable in three different vigilant attitudes, i.e., (a) vigilant lying, (b) vigilant standing and (c) vigilant moving; the three vigilant attitudes are described in Table 2. The three vigilant attitudes may give an insight into the intensity of the alarm. The animals are more relaxed when lying down, while they are more sensitive to disturbances when standing and moving [18]. 

Reactions to disturbances were recorded in 30 min observation sessions; they were distributed from 9 am to 6 pm depending on the presence of tourists.

At least one observation session was performed before, during and after the exposure to tourists per day. Different observation sessions were performed for the nursery and the male groups. When possible, an observation session for every time slot (before, during and after) was performed for both groups, depending on the arrival of tourists and the possibility of observing the animals, since the enclosure offers good hiding spots. During every observation session, all the stimuli were presented to the animals in random order. A detailed list of observation sessions is reported in Table S1 in the Supplementary Materials.

Responses were observed after presenting each stimulus; the next stimulus was presented only after the group stopped showing alarm reactions. Alarm reactions were counted and their intensities were recorded and divided into 3 levels of intensity depending on the transition from one vigilant attitude to another: Level 1, the animal showed an alarm signal but did not change its vigilant attitude; Level 2, the animal moved from a vigilant attitude to a more reactive one (from lying to standing, from standing to moving); Level 3, the animal ran away. The stimuli were presented both outside and inside the enclosure to test the difference in the number of alarm reactions and alarm levels. 

The experiment only started when the animals showed no alarm signals and were in a relaxed state. When entering the enclosure, the observer walked first beside the fence. This was important because the sound of the gate could alarm the animals. In this way, the animals were able to return to a relaxed state before starting testing with the stimuli inside the enclosure. Only when the animals were relaxed did the observer start moving toward them and presenting the stimuli. One observer always remained outside the enclosure. The time of day was recorded, as well as the time with respect to exposure to tourists (before, during, after). The visibility conditions were recorded as good or bad depending on the presence/absence of rain or fog.

### 2.3. Statistical Analysis

Data were analyzed in R (Version R4.1.2) [19]. A preliminary model, using Generalized Linear Mixed Models (GLMM, lme4 package [20]), was used to test the influence of the variables of observation session ID and group size as random effects. Marginal and conditional coefficients of determination were calculated (using r.squaredGLMM, MuMIn package), and no difference was found between the two. For this reason, the variables were excluded as they did not explain the variance in the data. A simpler generalized linear model (GLM, lme4 package) was therefore run without random factors; the comparison of the two models based on the AIC value confirmed the preference for the simpler model. The variable of visibility conditions was initially included in the model but was then excluded during the variable selection procedure. Based on the descriptive statistics, the variable groups that induced a lower average number of alarm reactions (Wednesday, before, afternoon, males, outside, standing) were included in the intercept as a baseline.

### 2.4. Effects of Disturbance on the Number of Alarm Reactions

Since the dependent variable (sum of alarm reactions i–iv) comprised count data, data were analyzed with Generalized Linear Models (GLM) using a Poisson link function to test for the effects of the various explanatory variables on the total number of alarm reactions shown by animals. A full model (with variables and their interactions) and a reduced model were produced (using the drop1 function) and selected based on their AIC values. Significance was set at *p* < 0.05. A post hoc pairwise comparisons test was then run using the glht function (Multcomp package, R, version R4.1.2). 

To understand which variable significantly affected the number of alarm reactions, seven variables were tested as fixed effects: type of stimulus (see Table 1), whether the stimulus was presented inside or outside (inside/outside), day of the week (day), time of the day (morning/afternoon), group type (nursery group or males), exposure to tourists (before, during or after tourists’ presence) and the interaction between the type of stimulus and inside/outside. 

### 2.5. Effects of Disturbance on the Intensity of Alarm Reactions

To understand which variables significantly affected the number of responses by the animals pertaining to the three levels of intensity, canonical redundancy analysis (RDA) [21] on the “stimulus x number of alarm reactions x level of intensity” matrix was performed, using the above-mentioned fixed effects as constraining variables (rda function of vegan R package, version 2.6-2). RDA is an extension of regression analysis that allows us to analyze the relations between multivariate response data (response intensity of animals in our study) and an explanatory variable data set. A preliminary backward selection of the explanatory variables and their interactions was performed starting from the full model using the ordistep function (vegan package) and then checking the reduced model to detect possible collinearities among the explanatory variables using the vif.cca function. No collinear variables were found. The significance of the reduced model was tested using the anova.cca function (vegan package), as well as axes’ and terms’ significance using, respectively, the by=“axis” and by=“term” arguments and running 999 permutations per test. The adjusted R-squared value of the model was obtained by using the function RsquareAdj.

## 3. Results

### 3.1. Behavioral Overview

**Figure 5:**
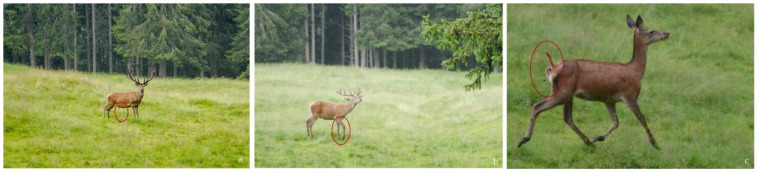
Three different alarm signals. (**a**) The urination before flight displayed by the dominant male. (**b**) The stomping of the hoof on the ground performed by a subordinate male. (**c**) The defecation during flight performed by a female without a fawn.

During the observation sessions, other unexpected alarm signals were observed; they were not included in the statistical analysis, but were very useful to better understand the animal behavior, so we decided to include them in this subsection. Such signals were performed by one animal in response to a higher disturbance and were probably aimed at alerting the whole group to the danger. The signals that we could observe were as follows:

- Urination before flight (Figure 5a);
- The stamping of the hoof on the ground (Figure 5b);
- Defecation during flight (Figure 5c).

**Figure 6:**
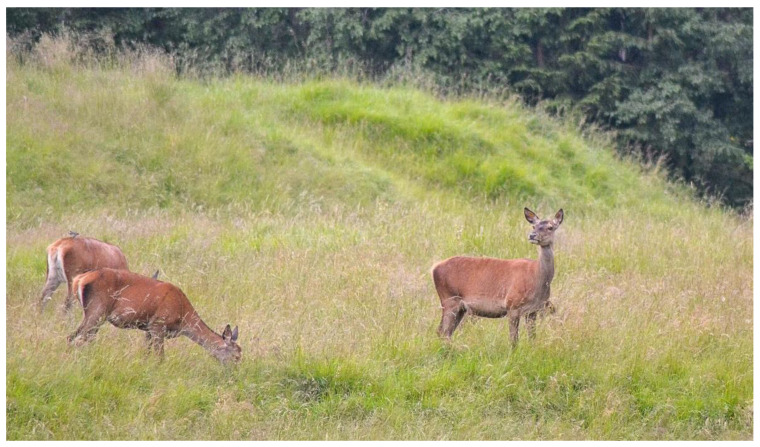
A female sentinel remains in an alert state while the other females eat.

Such signals were normally performed by the dominant male or by a female sentinel, a female without fawns in the nursery group, which remains always in an alert state, and was ready to warn the group of an approaching danger (Figure 6).

**Figure 7:**
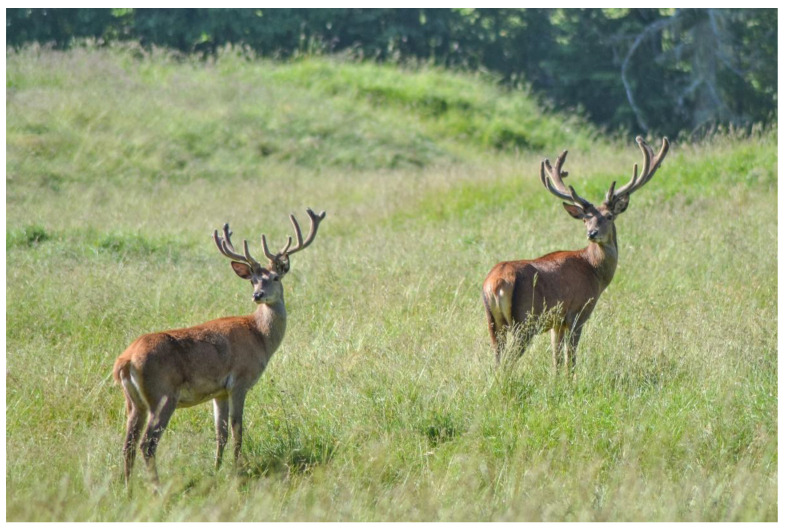
Two males stopping and looking back to the source of disturbance while fleeing.

Males and females had completely different reactions to disturbances. With good visibility conditions, the closest approach distance inside the enclosure to the males’ group was in fact ca. 7 m, while that to the nursery group was ca. 60 m; distances were recorded with a measuring tape after the observation sessions. The two groups also had different flight strategies. The males tended to frequently stop during the flight to check the disturbance source (Figure 7), while the nursery group, after initiating a flight, ran away immediately without stopping until a safe spot was reached. Moreover, other differences within the groups were noticeable. In the male group, the last one fleeing was always the dominant one, which was also the least sensitive to disturbances. In the nursery group, the females without fawns were less sensitive to disturbances. 

With poor visibility conditions, animals were less sensitive to disturbances. During a rainy day, in fact, the distance between the nursery group and the observer was ca. 30 m. Moreover, animals were less disturbed by known stimuli, even when close, while new stimuli alarmed the animals even when far from the enclosure. However, they tended to become habituated to particular stimulus after a few expositions. It was observed that deer perceived the enclosure as a defensive limit that tourists could not cross, but did not discern whether a person, if close to the enclosure, was inside or outside. This was also observed when entering the enclosure: if the observer walked beside the fence, the animals quickly went back to a relaxed state after their first reaction to the gate sound. When the observer started walking toward the animals, they started displaying increasing alarm reactions. A brief video of the described observation is available in the Supplementary Materials (Video S1).

### 3.2. Descriptive Statistics

A total of 27 observation sessions were analyzed. The mean number of alarm reactions per observation session was 9.07. During the weekend and on Monday, the animals were more sensitive to the stimuli; in fact, the mean number of alarm reactions was the highest on Monday (mean = 15.6; interquartile range (IQR) = 14.0) and Sunday (mean = 9.7, IQR = 5.0). Instead, the deer were less sensitive on Wednesday (mean = 6.2; IQR = 4). Exposure to tourists affected the animals’ reactions to disturbances. They were particularly reactive to disturbances during the exposure (mean = 11.5; IQR = 7.3), compared to before (mean = 6.9; IQR = 4). The nursery group was more easily alarmed (mean = 12.4; IQR = 8) compared to the males (mean = 6.7; IQR = 4). The type of stimulus produced visible differences. All the stimuli performed inside the enclosure induced higher alarm responses, and stimulus M produced the highest number of reactions (mean = 11.7, IQR = 9), almost double compared to stimulus S (mean = 6.8, IQR = 5.5). We did not observe a remarkable difference between morning and afternoon, but the stimuli presented in the morning alarmed the animals more (mean = 9.2, IQR = 8) compared to the stimuli presented in the afternoon (mean = 8.9, IQR = 6.7). The stimuli presented inside the enclosure (mean = 13.5, IQR = 10.5) produced more than the double the alarm reactions compared to the stimuli presented outside the enclosure (mean = 5.9, IQR = 4). The results of the descriptive statistics of the effects of key variables on the number of alarm reactions are summarized in Table 3.

### 3.3. Effects of Disturbance on the Number of Alarm Reactions

In order to test for the effect of several variables on the number of alarm reactions shown by the animals, GLM were used. The reduced model was selected on the basis of the AIC value. Stimulus U had a higher impact on the number of alarm reactions compared to S (*p* < 0.001) and to M (*n.s*.), while stimulus S had a lower effect than the other two types of stimuli (*p* < 0.001). The difference between morning and afternoon was not significant. The number of alarm reactions recorded on Monday was significantly higher than on Friday (*p* = 0.005), Wednesday (*p* < 0.001), Tuesday (*p* < 0.001) and Thursday (*p* = 0.002) and it was slightly significant on Saturday (*p* = 0.05) and Sunday (*p* = 0.07). On Wednesday, a lower number of alarm reactions was recorded, compared to Friday (*p* = 0.02), Saturday (*p* < 0.001), Sunday (*p* < 0.001), Thursday (*n.s*.) and Tuesday (*n.s*.). On Sunday, a significantly higher number of alarm reactions was recorded compared to Tuesday (*p* = 0.02). The tourists’ presence affected the deer’s reactivity; in fact, during the tourists’ presence, the animals showed a higher number of alarm reactions compared to before (*p* < 0.001) and to after (*n.s.).* After the tourists’ passage, the animals remained more reactive than before (*n.s.*). The two groups reacted to stimuli in different ways; in fact, the nursery group showed a significantly higher number of alarm reactions compared to the males (*p* < 0.001). The animals showed a higher number of alarm reactions when the stimuli were presented inside the enclosure (*p* < 0.001). Stimulus S presented outside significantly produced the lowest impact on the animals’ alarm reactions. The deer in fact presented a lower number of alarm reactions to it compared to all the stimuli presented inside S (*p* < 0.001), M (*p* < 0.001) and U (*p* < 0.001), and the other stimuli presented outside the enclosure, M (*p* = 0.009) and U (*p* = 0.004). Stimulus M presented inside had the highest impact on the number of alarm behaviors, significantly higher than stimulus S presented outside (*p* < 0.001), stimulus S presented inside the enclosure (*p* < 0.001), stimulus M presented outside (*p* < 0.001) and stimulus U presented outside (*p* < 0.001). GLM results are shown in Table 4, while the respective pairwise comparisons are shown in Table S2 in the Supplementary Materials.

### 3.4. Effects of Disturbance on the Intensity of Alarm Reactions

**Figure 8:**
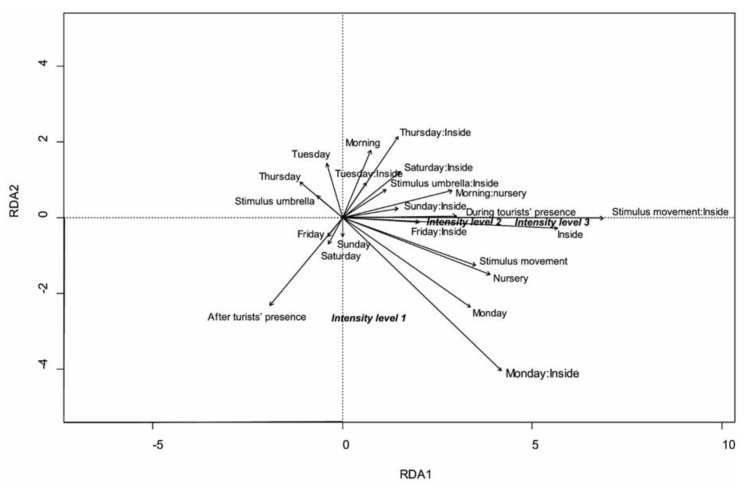
Redundancy analysis graph of the matrix “stimulus × number of animals’ responses per level of intensity”, using the following variables: type of stimulus, inside/outside, day of the week, time of the day (morning/afternoon), group type (nursery group or males), exposure to tourists (before, during or after tourists’ presence) and the interaction between the type of stimulus and inside/outside.

The RDA model explained globally 46% (adj. R-squared, *p* = 0.001) of the variance in the number of alarm reactions per level of intensity. The RDA graph (Figure 8) depicted a main gradient along axis 1, related to the number of alarm reactions and the intensity of the response, with the maximum number and intensity linked to the positive values. The variables that most positively responded to the number and intensity of reactions were as follows: during the tourists’ presence; the position inside the enclosure (inside) and its interactions with the type of stimulus (stimulus M) and with the days of the week (Friday and, secondly, Sunday); and the reaction displayed by the nursery group, along with its interaction with time (morning). The least intense reaction (intensity level 1) was mostly displayed after the exposure to tourists and on Friday, Saturday and Sunday.

## 4. Discussion

The results of this study suggest that the red deer adversely reacted to the disturbance by tourists to different degrees depending on the day of the week. Animals showed greater sensitivity to disturbances when many tourists were present; the higher number of alarm reactions and the higher alarm intensity observed indicated higher discomfort. We observed that the days with more tourist arrivals (Friday, Saturday and Sunday) led to higher alarm rates of animals, presenting a higher number of alarm reactions, as highlighted by the GLM results. During these days, the stimuli presented inside the enclosure induced a more intense response than during the other days, suggesting that the animals were disturbed to the point of changing their current state (changing attitude or fleeing) than on every other day. This is visible in the RDA graphs, where “Friday:inside”, “Sunday:inside” and “Saturday:inside” are positively related to the two highest intensity levels (2 and 3). However, as the GLM indicates, the highest number of alarm reactions was seen on Monday; this suggests that stressors’ effects build up during the weekend, leaving the deer particularly sensitive to disturbances, despite lower exposure to tourists. This is consistent with Sibbald et al. [22], who found that the disturbance effects were still visible after 24 h after exposure to hillwalkers. 

The disturbance effects’ accumulation may also explain the differences between the alarm reactions displayed before, during and after exposure to tourists. The animals were less sensitive to stimuli before tourists’ arrival and more sensitive during the tourists’ presence, with higher intensity during this particular condition. As shown in the GLM, the variable “during tourists’ presence” was significantly higher than previously seen and positively related to the alarm reaction intensity levels 2 and 3 in the RDA graph. Tourists induced a change in attitude in the deer, with possible negative effects on their welfare, derived from a change in time spent grazing, resting or nursing. The animals still appeared sensitive to the disturbance after the tourists had left, but with a lower intensity level (intensity level 1). 

The time of the day did not particularly affect the animals’ alarm reactions, but we observed a higher response on Monday morning. Stankowich’s study demonstrated that female deer and young animals appear to flee to greater distances than males [23]. Similarly, females with more vulnerable offspring exhibit greater flight responses than males and conspecific females [23]. In line with this, the nursery group of our study, which included mothers, dry females and fawns, was significantly more sensitive to disturbances compared to the group composed of males. Females in the nursery group were visibly less approachable than males, and showed a higher alarm level by quickly fleeing after being exposed to the stimulus, as shown in the RDA graph, with the variable “nursery group” positively related to intensity level 2 and 3. The distance of the closest approach to the male group was in fact ca. 7 m, while that to the nursery group was ca. 60 m with good visibility conditions. 

The results suggest that the deer responded differently to different visual stimuli. They were less reactive to stationary movements; a person standing (S) alarmed the animals less and with a lower intensity, while the movements toward the animals (M) produced a significantly higher number of alarm reactions at a higher intensity level, with the variable “stimulus M” positively related to alarm intensity 2 and 3. It would be more energy-efficient for the animal to remain alert to the sight of a predator but only react strongly (i.e., run away) when the predator approaches, being an imminent threat to the animal [18]. 

It was observed that deer perceived the enclosure fence as a form of protection; in fact, the stimuli performed outside the enclosure alarmed the animals less than the ones performed inside, with a lower number of alarm reactions and a lower intensity level. The stimulus M performed inside the enclosure particularly alarmed the deer, as shown in the GLM results and in the RDA graph, with the interaction “M:Inside” being the most related to alarm intensity 3. This is consistent with Whittington and Chamove’s study [24], where deer took longer to react to disturbances when offered shelter and after a habituation period. The same conditions were found in the population object of this study.

The animals were observed displaying excretion alarm signals, such as urination before flight and defecating during it. In other studies, ungulates were observed displaying the same alarm behaviors—for example, in goitered gazelle (*Gazella subgutturosa*) [25], pronghorn (*Antidorcas americana*) [26] and fallow deer (*Dama dama*) [27]. Generally, urination and/or defecation occurs as a response to stress in animals. Thus, excretion frequency can indeed be an index for fearfulness [27,28]. In this situation, the behaviors displayed may be aimed at alerting the group to an approaching danger, since, after the signal, all the other animals were observed fleeing. Experiments with cattle demonstrated that the urination of stressed individuals may increase the stress levels of other conspecifics, which in turn become more fearful [29]. Prolonged stress may lead to negative outcomes for the animal’s body [15] and, in trying to overcome such stress, the body will be placed in a survival state, and if it is not in a condition to cope with such a stressor, the animal will go through an exhaustion phase [30,31,32]. Prolonged and chronic stress is an established risk factor for the development of depression-like states [33], even when not visibly shown by the animal. 

We observed that the dominant male was particularly less sensitive to disturbances compared to the whole group of animals. However, as stated by Nemets et al. [15], dominants, even if resistant to transient stressors, are more subject to the actions of prolonged stress, with the subsequent development of a depression-like state. Inactive subordinates, susceptible to transient stressors, contrarily show greater resistance to prolonged disturbance, without negative effects on their psycho-emotional status [15]. Furthermore, clinical and preclinical studies have shown that females have higher sensitivity to stress [34,35]. 

It is crucial to avoid stress during crucial periods, such as gestation, birth and nursing; for this reason, it would be advisable to avoid carrying out any activity inside the enclosure in such periods (in the study case, from May to mid-July). Mating season (in the study case, September to October) may be dangerous for humans inside the enclosure, due to the high aggressivity displayed by males. In this particular location, it is not possible to work during the winter due to the snow cover. Therefore, for this study case, the best time slots to perform activities inside the enclosure are March and April (if snow is not present) and August. August, despite being the more suitable one, is of interest to a great number of tourists. Consequently, it is important to establish the days/times that may be particularly suitable, avoiding the accumulation of disturbances (high tourist presence, humans inside the enclosure, etc.), which may increase the negative effects on the animals’ welfare.

Based on the results obtained from data elaboration, we can conclude that the best day to perform activities inside the enclosure is Wednesday, being the day on which the red deer showed alarm reactions at a lower intensity level. In addition, the alarm reaction number observed on Wednesday was the lowest of all the other days, and significantly lower compared to the reactions observed on Friday, Saturday, Sunday and Monday. However, Tuesday and Thursday could also be suitable days to carry out activities inside the enclosure, considering that the number of alarm reactions was significantly lower than the one observed on Monday. Conversely, the days on which to avoid carrying out activities that may stress the animals, inside the enclosure, are Friday, Saturday, Sunday and Monday. For this study case, taking into consideration the functional area distribution inside the enclosure, it would be recommended to concentrate the required mowing actions of different areas on different days, allowing the entrance of a few operators each day, in order to ensure that the animals have the possibility of hiding in a safe place. Some measures may be helpful in controlling disturbances by tourists and preventing direct contact with the animals, taking into consideration some tourists’ inappropriate behavior, such as touching the more confident individuals through the fence or feeding the animals inappropriate food (even if not allowed). Building a second fence at least 2–3 m away from the principal deer enclosure might help to provide more space between the humans and the animals. In the study case, additional observation platforms should be constructed and placed so that visitors can view the animals from a respectful distance.

## 5. Conclusions

According to the study results, we can assert that the deer’s reactivity was affected by the tourists’ presence; a significantly higher number of alarm reactions were in fact observed during the tourists’ presence. The nursery group showed a significantly higher number of alarm reactions compared to the males. The animals showed a higher number of alarm reactions when the stimuli were presented inside the enclosure with respect to those presented outside. Finally, the days on which the animals were less sensitive and showed a lower number and intensity of alarm reactions were Wednesday, Tuesday and Thursday.

In order to buffer the negative effects on the deer’s well-being, it would be opportune to plan any action inside the enclosure during the days on which the animals are less sensitive to disturbances.

Although it may be necessary for different purposes to enter into the enclosure, this certainly creates a certain level of disturbance for the animals. Therefore, it is necessary to carry out any action inside the enclosure when the disturbance does not further increase the state of discomfort and fear of the animals, which must be as free as possible from such conditions.

Despite the observation period being quite limited, the obtained results might serve as a baseline for more in depth and prolonged studies, as well as to enhance the knowledge about the species’ behavior. In addition, the proposed observational method could be applied to other locations and on other captive species, in order to identify the main behavioral patterns of animals and to establish the best times to carry out different activities inside the enclosures.

## Figures and Tables

**Table 1:** Visual stimuli presented to the deer.

Stimulus	Stimulus Description
S	A person standing still
M	A person moving toward the animals
U	An umbrella opened

**Table 2:** Types of vigilant attitudes.

Vigilant Attitude	Criteria
Vigilant lying	Body stretched on the ground with head held high; occasional turning of the head and ear twitching.
Vigilant standing	Standing still with either head held parallel to the body or high; neck prominent; sometimes freezing the posture with occasional ear twitching.
Vigilant moving	Rapid pacing (either brisk walking or running) with head held high and neck very prominent and ears straight.

**Table 3:** Descriptive statistics, mean, median, Q1 (first quartile), Q3 (third quartile), IQR (interquartile range) of number of alarm reactions shown by deer as a reaction to the key variables. Alarm reactions before, after and during exposure to tourists, on Friday, Saturday, Sunday, Monday, Tuesday, Wednesday and Thursday, in the morning and in the afternoon, by males and nursery group, to the different stimuli, and to the stimuli presented inside or outside.

	Median	Mean	Q1	Q3	IQR
Before	6	6.929	4	8	4
After	8	8.242	4	10	6
During	9	11.5	8	15.25	7.25
Stimulus S	5	6.795	3.5	9	5.5
Stimulus M	9	11.66	7	16	9
Stimulus U	8	8.059	5	9	4
Morning	8	9.176	4	12	8
Afternoon	8	8.957	4	10.75	6.75
Males	5	6.754	4	8	4
Nursery	10.5	12.38	8	16	8
Friday	8	8.467	4	12.5	8.5
Saturday	8	9.231	6	9	3
Sunday	9	9.765	7	12	5
Monday	15	15.62	8	22	14
Tuesday	8	7.643	4	9.75	5.75
Wednesday	5	6.231	4	8	4
Thursday	4.5	6.333	3.75	8	4.25
Inside	12	13.05	8	18.5	10.5
Outside	5	5.907	4	8	4

Stimulus S = a person standing, stimulus U = an umbrella opened, stimulus M = a person moving toward the animals.

**Table 4:** Fixed effects table for the generalized linear model (GLM) fitted to the number of alarm reactions displayed by deer for type of stimulus, stimulus presented inside/outside the enclosure, time of the day, day of the week, exposure to tourists, group type and interaction between type of stimulus and inside/outside. Significance level set at *p* < 0.05.

	Estimate	Std. Error	z Value	Pr(>|z|)
(Intercept)	0.69492	0.25227	2.755	0.005876
Morning	0.07765	0.1531	0.507	0.612049
Friday	0.35715	0.15704	2.274	0.02295
Monday	0.72524	0.13959	5.196	<0.001
Saturday	0.48973	0.14639	3.345	<0.001
Sunday	0.51879	0.13895	3.733	<0.001
Thursday	0.22988	0.1802	1.276	0.202058
Tuesday	0.21046	0.1657	1.27	0.204053
After	0.28083	0.17769	1.58	0.114011
During	0.35799	0.10013	3.575	<0.001
Nursery	0.38549	0.08932	4.316	<0.001
Inside	0.58977	0.12993	4.539	<0.001
Stimulus M	0.44603	0.13289	3.356	<0.001
Stimulus U	0.56067	0.15675	3.577	<0.001
Inside:Stimulus M	0.10292	0.16405	0.627	0.530428
Inside:Stimulus U	−0.28722	0.21913	−1.311	0.189956

Stimulus U = an umbrella opened, stimulus M = a person moving toward the animals.

## Data Availability

Data supporting the reported results are kept in the archive of Sara Moscatelli.

## Supplementary Materials

The following supporting information can be downloaded at: https://www.mdpi.com/article/10.3390/ani13050903/s1, Table S1: List of observation sessions, Table S2: Tukey post hoc pairwise inter-group comparisons, Video S1: Alarm reactions of red deer.
