# Supplementary material for: Monitoring of Alarm Reactions of Red Deer (Cervus elaphus) in a Captive Population in Paneveggio Pale di San Martino Natural Park
Source: Animals (Basel). 2023 Mar 1;13(5):903. doi: 10.3390/ani13050903 (PMC10000108; doi:10.3390/ani13050903)
Supplement: Supplementary file 1 [file animals-13-00903-s001.zip › animals-2142126-supplementary materials.pdf]

Table S1. List of Observation sessions.

Observation Session ID	Daytime	Tourist Exposure	Group Type
Observation session 1	Morning	Before	Males
Observation session 2	Morning	During	Nursery
Observation session 3	Afternoon	After	Males
Observation session 4	Morning	Before	Nursery
Observation session 5	Morning	During	Males
Observation session 6	Morning	During	Nursery
Observation session 7	Afternoon	After	Nursery
Observation session 8	Afternoon	After	Males
Observation session 9	Morning	Before	Nursery
Observation session 10	Morning	During	Males
Observation session 11	Morning	During	Nursery
Observation session 12	Afternoon	After	Males
Observation session 13	Morning	Before	Males
Observation session 14	Morning	During	Males
Observation session 15	Afternoon	During	Nursery
Observation session 16	Afternoon	After	Males
Observation session 17	Morning	Before	Males
Observation session 18	Morning	During	Males
Observation session 19	Morning	During	Nursery
Observation session 20	Afternoon	After	Males
Observation session 21	Morning	Before	Males
Observation session 22	Afternoon	During	Nursery
Observation session 23	Afternoon	After	Males
Observation session 24	Morning	Before	Nursery
Observation session 25	Afternoon	During	Nursery
Observation session 26	Afternoon	During	Males
Observation session 27	Afternoon	After	Nursery

Table S2. Tukey Post hoc pairwise inter-group comparisons.

	Estimate	Std. Error	z value	Pr(>|z|)
M - S == 0	0.446	0.1329	3.356	<0.001
U - S == 0	0.5607	0.1568	3.577	<0.001
U - M == 0	0.1146	0.1435	0.799	0.424422
Morning - Afternoon == 0	0.07765	0.1531	0.507	0.612
Friday - Wednesday == 0	0.35715	0.15704	2.274	0.02295
Monday - Wednesday == 0	0.72524	0.13959	5.196	<0.001
Saturday - Wednesday == 0	0.48973	0.14639	3.345	<0.001
Sunday - Wednesday == 0	0.51879	0.13895	3.733	<0.001
Thursday - Wednesday == 0	0.22988	0.1802	1.276	0.202058
Tuesday - Wednesday == 0	0.21046	0.1657	1.27	0.204053
Monday - Friday == 0	0.36809	0.13195	2.79	0.005278
Saturday - Friday == 0	0.13258	0.14539	0.912	0.36182
Sunday - Friday == 0	0.16163	0.12977	1.246	0.212927
Thursday - Friday == 0	-0.12727	0.15018	-0.847	0.396736
Tuesday - Friday == 0	-0.14669	0.13719	-1.069	0.284949
Saturday - Monday == 0	-0.2355	0.12344	-1.908	0.056405
Sunday - Monday == 0	-0.20645	0.11785	-1.752	0.079817
Thursday - Monday == 0	-0.49536	0.16541	-2.995	0.002747
Tuesday - Monday == 0	-0.51478	0.14992	-3.434	<0.001
Sunday - Saturday == 0	0.02905	0.12891	0.225	0.821688
Thursday - Saturday == 0	-0.25985	0.16959	-1.532	0.12546
Tuesday - Saturday == 0	-0.27927	0.15704	-1.778	0.07534

Thursday - Sunday == 0	-0.2889	0.15607	-1.851	0.064146
Tuesday - Sunday == 0	-0.30833	0.13978	-2.206	0.027395
Tuesday - Thursday == 0	-0.01942	0.15217	-0.128	0.898436
After - Before == 0	0.28083	0.17769	1.58	0.11401
During - Before == 0	0.35799	0.10013	3.575	<0.001
During - After == 0	0.07716	0.14292	0.54	0.58929
NURSERY - MALES == 0	0.38549	0.08932	4.316	<0.001
Inside - Outside == 0	0.5898	0.1299	4.539	<0.001
Outside S- Inside S== 0	-0.58977	0.12993	-4.539	< 0.001
Outside S- Outside M == 0	-0.44603	0.13289	-3.356	0.00976
Outside S- Inside M == 0	-1.13871	0.11923	-9.551	< 0.001
Outside S- Outside U == 0	-0.56067	0.15675	-3.577	0.00441
Outside S- Inside U == 0	-0.86322	0.17099	-5.048	< 0.001
Inside S- Outside M == 0	0.14374	0.1156	1.243	0.8075
Inside S- Inside M == 0	-0.54894	0.09498	-5.779	< 0.001
Inside S- Outside U == 0	0.02909	0.14518	0.2	0.99995
Inside S- Inside U == 0	-0.27345	0.15556	-1.758	0.48083
Outside M - Inside M == 0	-0.69268	0.10453	-6.627	< 0.001
Outside M - Outside U == 0	-0.11465	0.14353	-0.799	0.96597
Outside M - Inside U == 0	-0.41719	0.15934	-2.618	0.08818
Inside M - Outside U == 0	0.57804	0.13681	4.225	< 0.001
Inside M - Inside U == 0	0.27549	0.14729	1.87	0.40822
Outside U - Inside U == 0	-0.30255	0.18078	-1.674	0.53705

U= An umbrella opened, M= A person moving toward the animals, S= A person standing still
